# Expression of p53 Protein Associates with Anti-PD-L1 Treatment Response on Human-Derived Xenograft Model of GATA3/CR5/6-Negative Recurrent Nonmuscular Invasive Bladder Urothelial Carcinoma

**DOI:** 10.3390/ijms22189856

**Published:** 2021-09-12

**Authors:** Ekaterina Blinova, Elena Samishina, Olga Deryabina, Dmitry Blinov, Dmitry Roshchin, Evgeniia Shich, Oxana Tumutolova, Ilya Fedoseykin, Anna Epishkina, Haydar Barakat, Andrey Kaprin, Kirill Zhandarov, Dmitrij Perepechin, Dmitrij Merinov, Gordey Brykin, Karen Arutiunian, Stanislav Serebrianyi, Artem Mirontsev, Andrew Kozdoba

**Affiliations:** 1 Department of Clinical Anatomy and Operative Surgery, Department of Pharmacology and Pharmaceutic Technology, Sechenov University, 8/1 Trubetzkaya Street, 119991 Moscow, Russia; bev-sechenov@mail.ru (E.B.); chih@mail.ru (E.S.); ilfedosei@yandex.ru (I.F.); afina-nn@mail.ru (A.E.); kirill-zhandarov@mail.ru (K.Z.); sechagord@mail.ru (G.B.); mirontsevsurgery@gmail.com (A.M.); 2 Department of Fundamental Medicine, National Research Nuclear University MEPHI, 31, Kashirskoe Highway, 115409 Moscow, Russia; 3 Laboratory of Molecular Pharmacology and Drug Design, Department of Pharmaceutical Chemistry, All-Union Research Center for Biological Active Compounds Safety, 23 Kirova Street, 142450 Staraja Kupavna, Russia; samy-elena@yandex.ru; 4 Laboratory of Pharmacology, Department of Pathology, National Research Ogarev Mordovia State University, 68 Bolshevistskaya Street, 430005 Saransk, Russia; dr.deryabina@gmail.com (O.D.); tumutol@rambler.ru (O.T.); 5 Laboratory of Molecular Pharmacology, Department of Clinical Trials and Scientific Research, Dmitry Rogachev National Medical Research Center of Pediatric Hematology, Oncology and Immunology, 1 Samory Mashela Street, 117997 Moscow, Russia; 6 Department of Oncological Urology, Russian National Research Medical Center of Radiology, Botkinsky Proezd, 125284 Moscow, Russia; dr89031990702@gmail.com (D.R.); bds131@yandex.ru (A.K.); medcraft@mail.ru (D.P.); d.merinov@gmail.com (D.M.); volecon@mail.ru (S.S.); 7 Department of Propaedeutic of Dental Diseases, People’s Friendship University of Russia, 6 Miklukho-Maklaya Street, 117198 Moscow, Russia; dr.haydarbarakat@yahoo.com; 8 Department of Urology, Andrology and Oncology, Pirogov Russian National Research Medical University, 1 Ostrovityanova Street, 117997 Moscow, Russia; dr.karen@mail.ru (K.A.); akozdoba@mail.ru (A.K.)

**Keywords:** nonmuscular invasive bladder cancer, p53 expression, double-negative molecular subtype, *FGFR3* mutations, FGFR3 expression, anti-PD-L1 therapy, human-derived xenograft model, mice, response

## Abstract

Background: The possible involvement of p53 signaling, FGFR3 expression, and *FGFR3* mutation rates in the prediction of the NMIBC anti-PD-L1 treatment response needs to be clarified. The main aim of our study was to explore predictive value of p53 expression, FGFR3 expression, and its gene mutation status for the therapeutic success of anti-PD-L1 treatment in the patient-derived murine model of recurrent high-PD-L1(+) GATA3(−)/CR5/6(−) high-grade and low-grade NMIBC. Methods: twenty lines of patient-derived xenografts (PDXs) of relapsed high-PD-L1(+) double-negative NMIBC were developed, of which 10 lines represented high-grade tumors and the other ones—low-grade bladder cancer. Acceptors of each grade-related branch received specific anti-PD-L1 antibodies. Animals’ survival, tumor-doubling time, and remote metastasis were followed during the post-interventional period. PD-L1, GATA3, CR5/6, and p53 protein expressions in engrafted tumors were assessed by immunohistochemistry. The FGFR3 expression and *FGFR3* mutations in codons 248 and 249 were detected by real-time polymerase chain reaction. Results: The expression of p53 protein is an independent factor affecting the animals’ survival time [HR = 0.036, *p* = 0.031] of anti-PD-L1-treated mice with low-grade high-PD-L1(+) double-negative NMIBC PDX. The FGFR3 expression and *FGFR3* mutation rate have no impact on the anti-PD-L1 treatment response in the interventional groups. Conclusions: p53 expression may be considered as a prognostic factor for the anti-PD-L1 treatment efficacy of low-grade high-PD-L1-positive GATA3(−)/CR5/6(−)-relapsed noninvasive bladder cancer.

## 1. Introduction

Bladder cancer (BC) remains the most common type of genitourinary malignancies worldwide, while urothelial carcinoma presents the majority of pathohistological forms of the bladder tumor. About 70–80% of all primarily detected bladder urothelial carcinomas initially develop without invasion into the muscular layer (pTa and pT1), and can be treated by transurethral resection [1,2]. At the same time, up to 70% of all cases tend to relapse, and about 25–30% of such cancers further demonstrate muscular-invasive behavior [3]. A high rate of nonmuscular invasive bladder cancer (MNIBC) recurrence and progression requires stringent follow-up procedures to be performed for each patient [4].

Clinicopathological features of BC such as clinical stage, tumor grade, and histology currently play a key role in progression risk assessment and making a decision for possible treatment options. However, none of them are considered as having strong prognostic value [5,6]. The molecular and genetic heterogeneity of NMIBC brings an additional input to the tumor behavior ambiguity. Thus, Dudhania et al. using a broad molecular panel demonstrated that GATA3-positivity strongly associated with the luminal molecular subtype of NMBIC, whereas CR5/6-positive carcinomas reflected the basal phenotype of the malignancy [7]. Along with double-negative NMIBC, they represent particular signaling, clinical, and prognostic patterns, which altogether determine the possible reaction to chemo-, immune-, and targeted therapy [7,8]. 

Cellular protein p53, one of the well-known tumor-inhibiting markers, regulates the cell cycle and is involved in cellular fate programming [9,10,11]. Mutations in the p53 protein-coding gene play pivotal roles in BC growth and progression [12]. On the other hand, before recently, there was a sufficient controversy in the comprehension of the prognostic meaning of the marker in BC [13,14]. Some investigators reported the beneficial role of p53 expression for the assessment of neoadjuvant chemotherapy, especially the DNA-targeted one [15,16,17]. However, the issue requires further clarification. Fibroblast growth factor receptor 3 (FGFR3) expression and its gene mutation rate are known to have a link with clinical outcomes in noninvasive bladder carcinoma [18]. *FGFR3* signaling downstream includes the activation of PI3K/AKT and RAS-MEK-ERK pathways, which accelerate tumorigenesis and associate with poor outcomes [19,20]. According to van Rhijn et al., *FGFR3* mutations have a driver role and are functionally distinct from FGFR3 overexpression [21]. Moreover, the low heterogeneity of *FGFR3* mutations determines a better prognosis for BC patients [22]. FGFR3 and p53 signaling interactions remain important points in cancer research. Nannapaneni et al. and Chen with co-authors showed that the co-expression of p53 and FGFR3 in oropharyngeal cancers associated with worse prognosis, whereas Mhavec-Fauceglia with colleagues found that NMIBC with the FGFR3(+)/p53(−) phenotype had distinctive meaning in tumorigenesis [23,24,25].

Development and clinical implementation of immune checkpoint inhibitors drastically changed the treatment landscape for many cancers, including BC [26,27]. Over recent decades, clinical and prognostic benefits of anti-PD-L1 treatment have been proved in a multitude of clinical trials and experimental studies [26,27,28]. It has also been shown that the response to immune- and biological therapy broadly varies depending on the molecular and histopathological type of BC, as well as the tumor microenvironment [29]. Taking into consideration the significant prognostic value of p53 signaling, the FGFR3 expression, and the *FGFR3* mutations rate in NMIBC, the possible involvement of the aforementioned biomarkers in the prediction of anti-PD-L1 treatment response needs to be clarified. Hence, the main aim of our study was to explore the predictive value of p53 and FGFR3 expression, and the *FGFR3* mutation status for the therapeutic success of anti-PD-L1 treatment in a patient-derived murine model of recurrent high-PD-L1(+) GATA3(−)/CR5/6(−) high-grade and low-grade NMIBC.

The design of the whole study according to Figure A3.

## 2. Results

**Figure A1:**
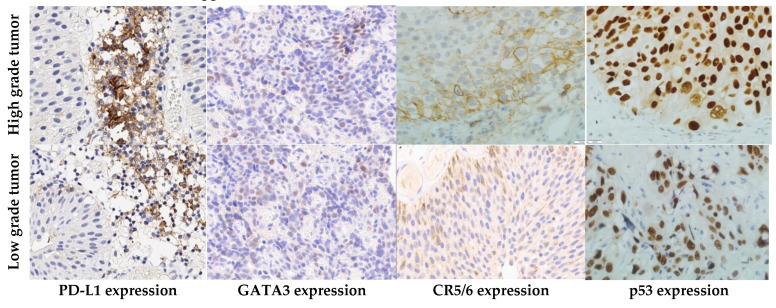
Immunohistochemical profile of the engrafted high- and low-grade double-negative noninvasive bladder tumors: anti-PD-L1, anti-GATA3, anti-CR5/6, and anti-p53 IHC staining, x500.

Twenty lines of patient-derived xenografts (PDXs) of relapsed high-PD-L1(+) GATA3/CR5/6-negative NMIBC were developed, of which 10 lines represented high-grade tumors and the other ones, low-grade bladder cancer. Figure A1 illustrates IHC patterns of the two main PDX branches referred to the tumor grade.

### 2.1. Biomarkers’ Expression Status in High- and Low-Grade PDXs

**Figure 1:**
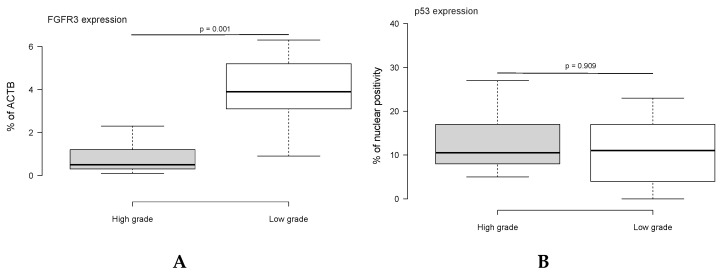
Expression of FGFR3 (**A**) and p53 (**B**) in high- and low-grade double-negative noninvasive recurrent bladder tumors engrafted into mice-acceptors; *n* = 10 in each group; median ± SD. The significance of differences between the groups was estimated by independent Student’s *t*-test.

Before intervention, the mean of FGFR3 expression in high-grade PDXs was 0.82, while, in low-grade tumors, it was an average of 3.85, with significant differences between groups (*p* = 0.001). The mean of p53-expressing cells before intervention in high- and low-grade tumors was 13.3 and 12.8, respectively. No significant differences were found between the groups (*p* = 0.909) (Figure 1).

**Figure A2:**
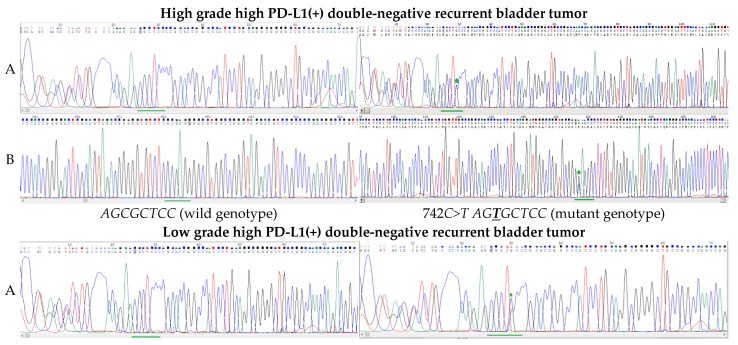

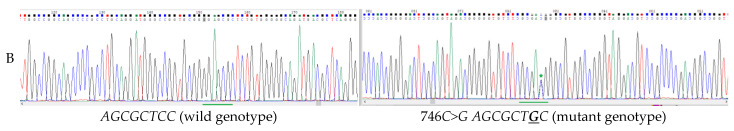
Hotspot mutations in codons 248 and 249 of *FGFR3* 7th exon in tumor cells of high- and low-grade double-negative NMIBC: (**A**) forward primer; (**B**) reverse primer; *AG**C**GCT**C**C* nucleotide sequence represents wild allele genotype; substitutions *C->T* in position 3 or *C->G* in position 7 referred to mutant *FGFR3* genotypes.

**Figure A3:**
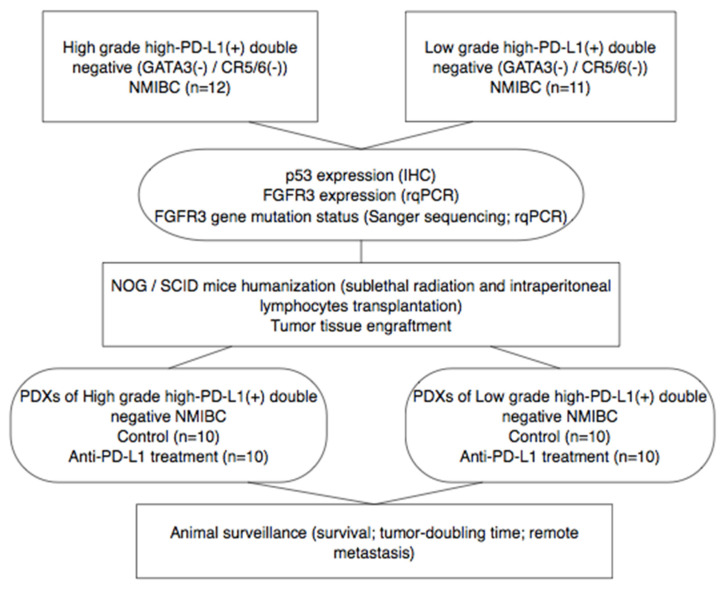
Study design.

*FGFR3* mutations (Figure A2) were found in 20% of high-grade tumors, while, in low-grade noninvasive BCs, the mutation rate was 60% with no significance in the intergroup comparison (*p* = 0.14, Mann–Whitney U-test). When assessing the p53 expression in terms of positivity and negativity according to a 10% cutoff, no statistical differences were found between high- and low-grade PDX groups.

### 2.2. Tumor Response to Anti-PD-L1 Experimental Therapy

Table 1 contains detailed information of the particularities of the tumors’ response to the anti-PD-L1 experimental treatment in control and interventional groups. The tumor-doubling time in high-grade and low-grade controls significantly differed and averaged at 9.8 and 17.2 days, respectively (*p* = 0.001). Interestingly, in appropriate interventional groups, the variable values were 18.3 ± 2.1 and 21.7 ± 2.1, respectively (*p* > 0.05). Anti-PD-L1 therapy prolonged the tumor-doubling time to 18.3 days vs. 9.8 in the appropriate control (*p* = 0.001), whereas we did not find a significant increase in the index value in the low-grade treatment group. The intergroup comparison revealed a decrease in the number of remote metastasis in the low-grade control—46.1 ± 2.3 vs. 69.3 ± 3.9 in the high-grade one (*p* = 0.003). In both interventional groups, a significant anti-metastatic effect was registered when compared with appropriate controls (*p* = 0.003).

**Figure 2:**
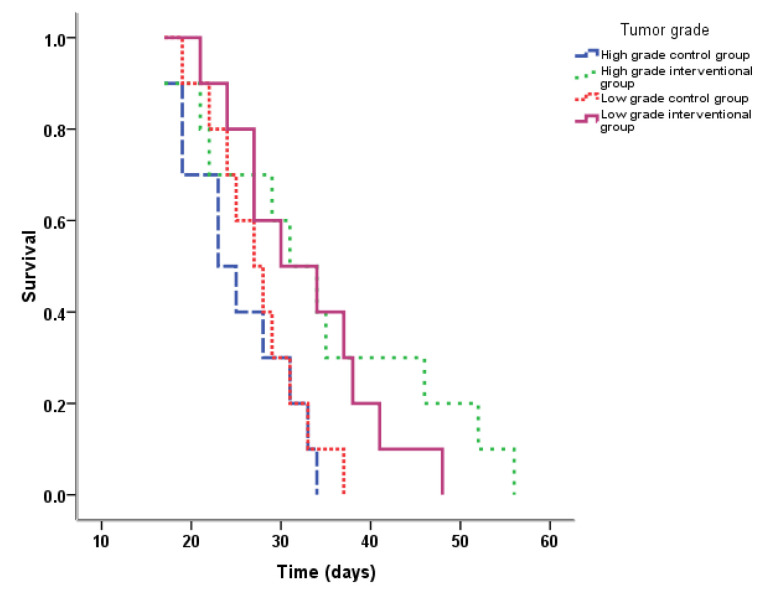
Kaplan–Meier plot for cumulative survival of mice carrying high- and low-grade NMIBC in control and interventional groups.

For animals’ survival time among the study groups, the Kaplan–Meier actuarial analysis showed that the median time was lowest in the low-grade interventional group (Figure 2). According to log-rank test, there was a significant difference in survival time among the study groups (*p* < 0.05).

**Figure 3:**
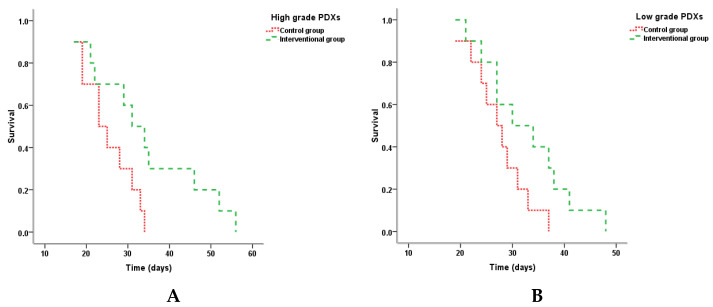
Kaplan–Meier plots for cumulative survival of animals, PDX carriers, in high- (**A**) and low- (**B**) grade NMIBC control and interventional groups.

The median of survival time in the high-grade NMIBC interventional group was 31 days, which was significantly higher than that in the control (23 days) (*p* = 0.043). When assessing the survival time in the groups of low-grade PDXs acceptors, no statistical differences were found between the control and interventional groups (*p* = 0.072) (Table 2, Figure 3).

### 2.3. FGFR3 Gene Mutations, and p53 Expression and Response to Anti-PD-L1 Therapy

**Figure 4:**
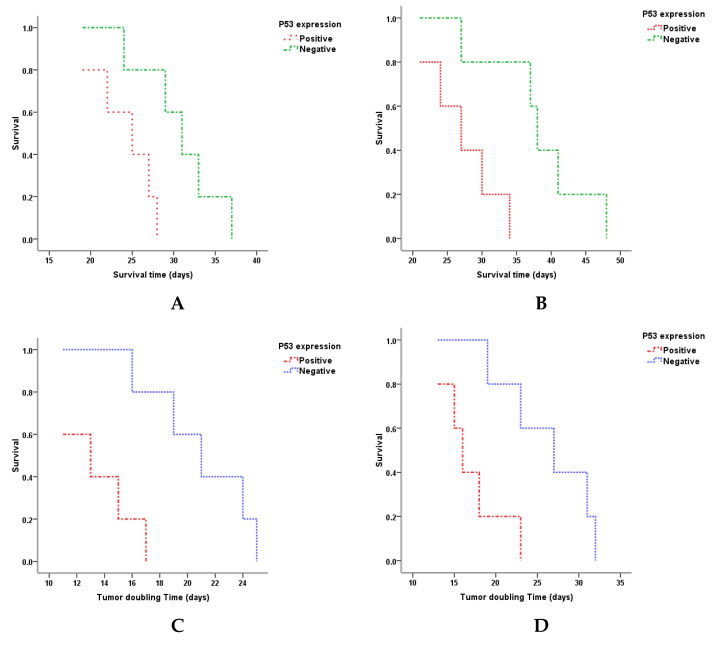
Kaplan–Meier actuarial analysis for animals’ survival time (plots (**A**,**B**)) and tumor-doubling time (plots (**C**,**D**)) in association with p53 protein expression in low-grade NMIBC PDX tumors: (**A**,**C**) represent the control, while (**B**,**D**) represent the interventional groups.

For the association of *FGFR3* mutations, p53 protein expression, and tumor doubling time, metastasis, and survival time, we used Kaplan–Meier analysis with the single-factor log-rank test. We found no differences in the association between *FGFR3* mutations and the studied variables in both control and interventional groups. In the control group with low-grade noninvasive double-negative bladder carcinoma, differences were found in the association of p53 expression and tumor-doubling time (*p* = 0.006) and survival time (*p* = 0.02) (Table 3, Figure 4). In the interventional group with the low-grade tumor, differences were found in the association of p53 expression and tumor-doubling time (*p* = 0.014), and animals’ survival time (*p* = 0.014) (Table 3, Figure 4).

### 2.4. Subsection

Three factors (FGFR3 expression, *FGFR3* mutations, and p53 protein expression) among the control and interventional groups with high- and low-grade bladder tumor were included in the univariate analysis using the Cox univariate regression model. It turned out that p53 protein expression in the control group with low-grade double-negative high-PD-L1(+) NMIBC [HR = 0.037, *p* = 0.033] and in the appropriate interventional group [HR = 0.036, *p* = 0.031] was the independent factor affecting the animals’ survival time. (Table 4). The other factors, such as FGFR3 expression and its gene mutation rate, were not associated with the animals’ survival time and tumor-doubling time in the experimental groups (*p* ˃ 0.05). We found that tumor grade was associated with both animals’ survival time and tumor-doubling time ([HR = 6.718, *p* = 0.015] and [HR = 0.387, *p* =0.009], respectively).

## 3. Discussion

Bladder cancer represents one of the most widespread types of malignancies. The genetic heterogeneity of the disease along with the complexity of its signaling results in the tumor variative behavior and clinical features. Intrusion into the muscular layer is considered a pivotal point of urothelial carcinoma evolution, determining the further development, outcomes, and prognosis. The current spectrum of treatment approaches based on molecular, genetic, clinical, and pathological features of the disease includes surgical, radiological, and therapeutic ones, as well as their combination. At the same time, recent advances in translational oncology have made it possible to implement principles of personalization regarding almost all kinds, even the most severe, of malignant neoplasm. The prior decade brought novel therapeutic strategies in oncology, revealing the high potency of immune checkpoint inhibitors in the control for the progression of many cancers, of which bladder carcinoma was not an exception. However, the first euphoria of treatment success has gradually been substituted by the comprehension of the therapy limitations, associated with sophisticated signaling interactions within the tumor cell. This insight was an incentive for the initiation of a study that would explain the possible involvement of well-known BC prognostic markers, such as p53 protein expression, FGFR3 expression, and its gene mutations status, in the prediction of tumor response to specific anti-PD-L1 therapy.

To do so, a PDX animal model was established. We based the current research on our previous experience of PD-L1-expressing NMIBC xenograft modeling [30,31,32]. Animals’ humanization includes sublethal irradiation and lymphocytes transplantation. We focused on the so-called double-negative noninvasive recurrent bladder carcinoma due to its poor clinical prognosis [33]. Moreover, previous studies have shown the prevalence of PD-L1-expressing CD8+ cells in the GATA3/CR5/6-negative immune microenvironment along with the moderate to high expression of the biomarker by tumor cells [29]. All PDX lines were initially divided into high- and low-grade, and for achieving equality regarding further treatment options at baseline, only high-PD-L1-expressing samples were selected. At the same time, it should be underlined that, though the heterotopic way of the tumor engraftment is suitable for the transplanted cancer growth assessment, the localization of the tumor in the bladder represents the natural course of the disease development. In our case, we chose the heterotopic one for the precise evaluation of tumor response to anti-PD-L1 treatment.

The initial molecular evaluation of patient-derived tumor samples was characterized by higher FGFR3 expression in low-grade tumor lines in comparison with high-grade ones, and an equal intergroup level of both p53 protein expression and *FGFR3* mutation rate. The obtained results were generally consistent with previously reported data of FGFR3 profile differences for high- and low-grade BCs [18,19,20,21,22]. The absence of differences between BC PDXs grades in terms of p53 expression and *FGFR3* mutation status rather supported those who thought them as having no predictive value [13,14].

The experimental treatment with anti-PD-L1 antibody Durvalumab resulted in the significant prolongation of survival time in a group of humanized NOG/SCID mice-accepted high-grade bladder tumors. The effect was accompanied by the increase in tumor-doubling time and inhibition of metastatic activity. Despite the low-grade tumor PDXs behaving less aggressively—animals’ survival and tumor-doubling time overcame both variables for high-grade BCs in the appropriate control group—and had a similar PD-L1-expressing status, they did not respond to the therapy. Therefore, using Cox regression, we found out that tumor grade possessed an independent predictive value for anti-PD-L1 treatment efficacy.

Analysis of the interaction between variables of the treatment response and studied biomarkers demonstrated controversial results. First, there were neither any link among survival time and tumor-doubling time, and FGFR3 expression, *FGFR3* mutations rate, and p53 expression in the control group nor in the interventional group with high-grade double-negative recurrent BC PDXs. In contrast, though low-grade PDXs did not respond to specific intervention, we determined that p53 expression interacted with the animals’ survival time in both low-grade groups. Similar results were demonstrated by the research team from the MD Anderson Cancer Center, who found the upregulation of the *TP53* gene in so-called p53-like invasive bladder tumors of 73 MIBC patients with poly-chemo-resistance to all kinds of neoadjuvant therapy [8]. Therefore, the “therapeutic behavior” of p53-expressing low-grade recurrent GATA3/CR5/6-negative noninvasive bladder carcinoma may have a p53-like MIBC-like pattern. The obtained results logically reveal some important questions as follows: whether the particularities of the previous intervention targeted primary-diagnosed bladder tumor in patients whose tissues were used in PDX modeling, have an impact on further experimental anti-PD-L1 therapy; whether there is any link between the aforementioned biomarkers and clinical outcomes in low- and high-grade cohorts of patients with recurrent double-negative p53-expressing NMIBC regarding treatment options. All those concerns need further clarification and will be the focus of our research.

## 4. Materials and Methods

### 4.1. Ethics Statement

The animal study met all requirements of international regulations. The study protocol was reviewed and approved at the joint meeting of the Ethics Committee of Sechenov University (Moscow, Russia) and Independent Bio-Ethic Committee of National Research Ogarev Mordovia State University (Saransk, Russia) on 9 April 2020 (No. of Approval Report 9—5 April 2020). The possibility of using human tissues for engraftment was discussed and reviewed by the Scientific Board of the Russian National Research Medical Center of Radiology (Moscow, Russia). Approval of the Center Ethics Committee (No. 07/05/12-2020) was received on 12 May 2020.

### 4.2. Study Design

A schematic of the study design is shown in Figure 1B. Fresh tumor tissue samples of the bladder tumor were collected from 23 patients with detected relapse of high-PD-L1(+) GATA3(−)/CR5/6(−) NMIBC, who underwent cystoscopy with biopsy or transurethral tumor resection at the Department of Oncourology of the Russian National Research Medical Center of Radiology from June to October, 2020; 11 samples represented recurrent low-grade noninvasive bladder carcinoma, while the other ones met clinicopathological criteria of high-grade NMIBC. Each sample was used for the development of a single heterotopic xenograft line in humanized NOG/SCID mice. Initially, resected tumor tissue was divided into three pieces, of which the first, fresh one was transplanted, the second piece was fixed and used in histological examination (HE), and the third fragment was frozen and further processed for immunohistochemistry (IHC), and molecular and genetic analysis. At the final stage of the xenograft establishment, each tumor line was subcutaneously inoculated into two mice, one of which was assigned to the control group, and the other to the intervention group. Four experimental groups, comprising 10 mice, tumor-bearing in each one, were made up for intervention and surveillance: high-grade MNIBC control and intervention; low-grade recurrent noninvasive bladder carcinoma control and intervention. Animals of the intervention groups received anti-PD-L1 therapy, and the mice of control groups were sham-treated with phosphate-buffered saline (PBS). FGFR3 expression, *FGFR3* hotspot mutations, and p53 expression were assessed in each tumor sample. For every animal, we registered the tumor-doubling time, survival time, and, after autopsy, the presence and number of lung metastasis.

### 4.3. Tumor Sample Sources

Tumor tissues were surgically removed from 23 patients, who underwent cystoscopy with biopsy or transurethral tumor resection from June to October, 2020. All patients, among whom there were 10 females of 64.8 ± 1.8 years old and 13 males of average age 62.3 ± 2.7, were properly informed about the study design and experimental protocol, and a consent form for tissue processing was voluntarily given in accordance with standard procedures. Table A1 contains the patients’ main gender characteristic and clinicopathological features of the tumors. The preliminary cancer sample selection was based on inclusion and exclusion criteria, and comprised clinical assessment, case history analysis, HE, and IHC.

Inclusion criteria covered adults (aged 18 and older) with relapsed low- and high-grade NMBIC-expressed high-PD-L1(+) with GATA3 and CR5/6 negativity. The muscular invasive type of BC, cases with urinary infection, and poorly processed tissues were excluded from the study.

Histologically, the majority of bladder tumors represented urothelial papillary carcinoma, and three cases were referred as micropapillary carcinoma and squamous cancer. Tumor grade and individual risk were assessed according to EORTC risk tables [1] and the WHO classification [30]. The previous treatment of patients with primary-diagnosed low-grade BC included intravesical Mitomycin; high-grade NMIBC patients received immunotherapy of Bacillus Calmette-Guerin (BCG) intravesically.

### 4.4. Laboratory Animals and Xenograft Modeling

For engraftment, we used 6–8-week-old immunodeficient NOG/SCID mice of both sexes. Laboratory animals were purchased at the Specific pathogen-free (SPF) Animals Breeding Facility of the Shemyakin and Ovchinnikov Institute of Bioorganic Chemistry of the Russian Academy of Sciences (Moscow Region, Russia). Mice were kept separately under sterile conditions in individually ventilated polyurethane cages at the Animal facility of Pharmacology and Pharmaceutic Technology Department of Sechenov University. Pathogen-free water and autoclaved standard food were provided ad libitum. Animals were housed on natural day/night cycles; room temperature at 25 ± 2 °C and humidity at 60 ± 5% were maintained in each cage.

When establishing the xenografts, we based them on previously described approaches for the heterotopic engraftment of bladder carcinomas [31,32,34]. All animals underwent humanization before tumor transplantation. The procedure included subsequent sublethal irradiation by 3.5 Gy at 0.8 Gy/mn (“Roentgen-TA 150/10” for radiotherapy, SpektrAP Ltd., Moscow, Russia) and then, after 3 days, the intraperitoneal transplantation of human lymphocytes approximately 5 × 10^7^ cells/1 mL/mouse. Human whole blood was obtained from healthy donors’ blood packs at the Blood Preservation Department of Dmitry Rogachev National Medical Research Center of Pediatric Hematology, Oncology and Immunology (Moscow, Russia). To isolate lymphocytes, whole blood was centrifuged with density gradient medium Lymphoprep^TM^ (Progene, New York, NY, USA) at 1200× *g* during 20 min.

A fresh piece of approximately 5.0 mm^3^ of each tumor specimen designated to transplantation was cut into 8–10 small pieces about 0.6–0.8 mm^3^ in size by sterilized microsurgical scissors (World Precision Instruments, Sarasota, FL, USA), and then all these particles were engrafted subcutaneously into the dorsal thigh of an animal with assistance of a hypodermic reusable needle G14 (GPC Medical Ltd., New Delhi, India). After the engrafted mass expanded to over quadruple its size, the xenograft tumor was harvested and directly re-transplanted for expansion in later serial generations using the same procedure. After the tumor tissue was passaged three times and HE confirmed the PDX to be a growing human tumor, the PDX line of each subtype of bladder cancer was considered as “established.”

### 4.5. Experimental Intervention and Surveillance

By the interventional stage of the study, four experimental groups were made up. Animals with xenografts of low-grade high-PD-L1 expressing double-negative MNIBC designated in the control and intervention groups (n = 10 in each one) represented the first experimental branch. The second study branch comprised acceptors of high-grade high-PD-L1(+) GATA3(−)/CR5/6(−) noninvasive BC with a similar animals’ distribution between the appropriate control and interventional groups with 10 mice in each one. The experimental intervention started when the volume of xenograft malignant nodes, measured twice weekly, advanced to 100–150 mm^3^. Mice of interventional groups were injected twice with 118.0 mg/kg of Durvalumab (Imfinzi**^TM^**, AstraZeneca, Cambridge, UK) via a tail vein, using the dual programming syringe-pump Genie Touch**^TM^** (Kent Scientific, Torrington, CT, USA). The first administration was made at day 0, and the same dose was injected 4 weeks afterward. Mice of control groups received intravenously (IV) the same volume of PBS. The Durvalumab of both animal’s dose and regimen were calculated by taking into account available murine toxicity data, and information of the substance clinical efficacy and safety. To calculate the effective murine dose, we multiplied 10 mg/kg (medium effective dose for humans) by 11.8 (converting coefficient for mice) [35,36,37]. Animals’ surveillance followed the second injection and included daily tumor calipering. We used a previously described formula for tumor volume measurement [38,39]. Animal survival time (AST), tumor-doubling time (TDT), and occurrence and number of remote metastasis were used for complex assessment of the experimental treatment response. AST was defined as the number of days from the day of the treatment cessation until an animal’s natural death or euthanasia due to severe uncontrollable pain. The period required to double the initial tumor volume (200%), including day 0, was considered as TDT.

A facial scale model was used to control the pain reaction in all experimental groups [40,41]. Moderate pain was controlled with 100 mg/kg twice daily of Ketoprofen (substance K1751, Merck Sigma-Aldrich, Darmstadt, Germany, purity > 98%) administered intragastrically via a curved feeding needle (Kent Scientific, Torrington, CT, USA) [42,43]. Animals suffering severe pain uncontrolled with painkillers were euthanized. Postmortem examination included measurements of the primary tumor nodes and counting of remote superficially visible lung metastasis.

### 4.6. Immunohistochemistry

We used 4 µm-thick tissue sections of formalin-fixed and paraffin-embedded tissue samples for immunohistochemistry (IHC). Tumor tissues were processed automatically by a Spin vacuum processor STP250-V (Histo-Line Laboratories Srl, Pantigliato, Italy) according to the standard protocol.

For the initial selection of the GATA3(−)/CR5/6 (−) phenotype of recurrent noninvasive bladder cancer, we assessed the GATA3 and CR5/6 expression status using monoclonal anti-human GATA3 (clone HG3-31, 1:100 dilution; Santa Cruz Biotechnology Inc., Santa Cruz, CA, USA) and anti-human CR5/6 (clone D5/16B4, 1:50 dilution, Dako, Glostrup, Denmark) antibodies. The tumor double-negativity was defined as the simultaneous occurrence of low/undetectable (<10%) GATA3 nuclear positivity combining with a similar cutoff value for CR5/6 cellular cytoplasm staining [44,45]. IHC staining for p53 positivity assessment was performed on an Autostainer Link (Dako, Glostrup, Denmark) using mouse monoclonal anti-human p53 antibodies (clone DO-7, Dako, Glostrup, Denmark). More than 10% of nuclear positivity was considered as a p53-expressing slice [15].

To detect high-PD-L1(+) NMIBC BC samples, we assessed the PD-L1 expression automatically on a Ventana BenchMark ULTRA ICH stainer (Ventana Medical Systems, Inc., Tucson, AZ, USA), as per the manufacturer’s instructions. The Ventana PD-L1 (SP263) Assay with the OptiView DAB IHC Detection Kit (Cat. No. 760–700/06396500001) and signal amplification (Ventana Medical Systems, Inc., Tucson, AZ, USA) were used in accordance with the manufacturer’s protocol [45] and previous evidence of SP263 clone selectivity for the tumor tissue PD-L1 expression assessment [29,46,47]. The high-PD-L1-expressing status relied on more than 25% of membrane positivity of the tumor and/or immune cells (if the latter counted above 1% of total cell population) scored in five randomly chosen high-power fields [46].

All sections were assessed by two pathologists blindly and independently. Five cases, in which the reviewers disagreed, were reassessed by consensus.

### 4.7. Reverse Transcription Real-Time Polymerase Chain Reaction

After thawing of freshly frozen BC tissues, we homogenized them in 600 µL of RLT Plus buffer (Qiagen, Hilden, Germany) with a TissueLyser LT homogenizer (Qiagen, Hilden, Germany) and 1% beta-mercaptoethanol placed in Lysing Matrix A tubes (MP Biomedicals, Santa Ana, CA, USA). DNA and RNA were isolated with the assistance of the AllPrep DNA/RNA/miRNA Universal Kit (Qiagen, Hilden, Germany) in accordance with the manufacturer’s instructions. Nucleic acid concentrations were measured using the Qubit dsDNA HS Assay for DNA concentration measurement, and Qubit RNA BR Assay kits (Thermo Fisher Scientific, Waltham, MA, USA) for estimation of the RNA concentration on a “Qubit 4” fluorimeter (Thermo Fisher Scientific, Waltham, MA, USA).

For a sample preprocessing, 20 pm of a reverse transcription (RT) primer oligonucleotide was mixed with 100 ng of total RNA in 9 µL and then incubated for 2 min at 70 °C; after that, the mixture was chilled on ice. For *FGFR3* and *ACTB* expression experiments, we used random decamers listed below. RT was performed for 30 min at 42 °C using the MMLV RT kit (Evrogen, Moscow, Russia) as per the manufacturer’s recommendations. The reaction was stopped by incubating the reaction mixture with inactivating reverse transcriptase for 10 min at 70 °C. Then, 10-fold diluted cDNA was used for RNA expression analyses by qPCR. For *FGFR3* and *ACTB* expression assessment, real-time polymerase chain reaction (qPCR) experiments were performed using the “qPCRmix-HS SYBR” (Evrogen, Moscow, Russia) on a DT Prime amplifier (DNA technology, Moscow, Russia) as per the manufacturer’s recommendations. In brief, 4 pm of each PCR primer and 1.5 µL of cDNA solution were amplified in 25 µL according to the following protocol: (1) denaturation of DNA at 95 °C for 2 min; (2) 45 cycles of the following: denaturation of DNA at 95 °C for 10 s; primer annealing at 67 °C for 3 s; elongation at 72 °C for 18 s; (3) analysis of melting curve.

We used the following oligonucleotide primers: for FGFR3—FGFR3RT-F, 5′-CCCAAATGGGAGCTGTCTCG-3′; FGFR3RT-R, 5′-CATCTCAGACACCAGGTCCG-3; for ACTB—b-act-for, 5′-GAGCGGGAAATCGTGCGTGACATT-3′; b-act-rev; 5′-GATGGAGTTGAAGGTAGTTTCGTG-3′.

### 4.8. FGFR3 Gene Hotspot Mutations Detection

For detection of mutations in codons 248 and 249, PCR-amplification of *FGFR3* exon 7 was performed. For this, we used qPCRmix-HS (Evrogen, Moscow, Russia), 100 ng of genomic DNA, 10 pm of the primer oligonucleotides (*FGFR3-7F*, 5′-*AGTGGCGGTGGTGGTGAGGGAG*-3′; *FGFR3-7R*, 5′-*ACCTTGAGCACGGTAACGTAGGGTGT*-3′), and the following protocol: (1) denaturation of DNA for 3 min at 95 °C; (2) 32 cycles of the following: denaturation of DNA for 20 s at 95 °C; primer annealing for 20 s at 70 °C, and elongation for 20 s at 72 °C; (3) storage at 4 °C. Agarose electrophoresis and the column purification kit “Cleanup Standard” (Evrogen, Moscow, Russia) were used for chain PCR products purification. We used both *FGFR3-7F* and *FGFR3-7R* primers separately for purified chain reaction products sequencing by Sanger at Evrogen (Moscow, Russia). We used nonmodified oligonucleotide primers purchased at Evrogen (Moscow, Russia) and fluorescently labeled oligonucleotide probes purchased at DNA-Synthesis (Moscow, Russia). Obtained data were analyzed with Chromas 2.6.6 software (Technelysium, Brisbane, Australia).

### 4.9. Statistical Analysis

The statistical analysis was performed using the SPSS statistical software version 22.0 (SPSS, Inc., Chicago, IL, USA). Descriptive statistics was used for analyzing the factors before intervention. For comparison of the variables before intervention, an independent Student’s t-test and Mann–Whitney U test were used. For association between variables: FGFR3 expression, FGFR3 mutations, P53 expression, tumor grade and tumor double time, metastasis, and survival time, Kaplan–Meier curves were used, and significance was classified by the log-rank test. The Cox regression model was used for prognostic analysis. Significance of differences was set on *p* < 0.05.

## 5. Conclusions

Twenty lines of patient-derived xenografts (PDXs) of relapsed high-PD-L1(+) GATA3/CR5/6-negative NMIBC were developed, of which 10 lines represented high-grade tumors and the other ones—low-grade bladder cancer;

(a) Low-grade high-PD-L1(+) double-negative recurrent noninvasive bladder carcinoma was characterized by a higher FGFR3 expression than that of high-grade bladder tumor. There were no differences between high- and low-grade tumors in p53 protein expression and *FGFR3* mutation rate;
(b) Experimental anti-PD-L1 therapy with Durvalumab significantly increased animals’ survival time and tumor-doubling time, and inhibited metastatic activity in the group of high-grade high-PD-L1(+) GATA3/CR5/6-negative BC PDX acceptors, whereas it had no influence on the mentioned variables in the low-grade group except the number of remote metastasis;
(c) We found no link among FGFR3 expression, *FGFR3* mutations status, and animals’ survival time, tumor-doubling time, and metastatic activity in both high- and low-grade control and interventional groups;
(d) The expression of p53 protein was an independent factor affecting the animals’ survival time of anti-PD-L1-treated mice with low-grade high-PD-L1(+) double-negative NMIBC PDX.

## Figures and Tables

**Table 1:** PDX tumors’ response to experimental therapy with Durvalumab (n = 10 in each control and interventional group).

Experimental Group	Tumor-Doubling Time, Days M ± SD		Number of Lung Metastasis, M ± SD
High-Grade Double-Negative High-PD-L1(+) BC	C	9.8 ± 0.9	69.3 ± 3.9
	T	18.3 ± 2.1 ^‡^	35.3 ± 7.6 ^‡^
Low-Grade Double-Negative High-PD-L1(+) BC	C	17.2 ± 1.6 ^†^	46.1 ± 2.3 ^†^
	T	21.7 ± 2.1	22.4 ± 4.5 ^‡^

C—grade-related control group; T—mice treated with Durvalumab; ^†^ *p* < 0.05 when compared with high-grade control (independent Student’s *t*-test); ^‡^ *p* < 0.05 when compared with appropriate control (independent Student’s *t*-test).

**Table 2:** Log-rank test for animal survival in control and interventional groups.

Study Group	Estimated Time (Days)	Log-Rank Test	*p* Value
High-grade BC control	23	4.091	0.043
High-Grade BC Intervention	31		
Total	28		
Low-grade BC control	27	3.231	0.072
Low-Grade BC Intervention	30		
Total	28		

**Table 3:** Association between p53 protein expression and animals’ survival and tumor-doubling time in control and interventional groups (single-factor log-rank test).

Variable	p53 Protein Expression	Estimated Time (Days)	Log-Rank Test	*p* Value
High-grade NMIBC control group				
TDT	Positive	8	1.341	0.247
	Negative	11		
	Total	9		
ST	Positive	23	0.811	0.368
	Negative	28		
	Total	23		
High-grade NMIBC intervention group				
TDT	Positive	14	0.016	0.900
	Negative	19		
	Total	16		
ST	Positive	31	0.000	0.982
	Negative	35		
	Total	31		
Low-grade NMIBC control group				
TDT	Positive	13	7.477	0.006
	Negative	21		
	Total	16		
ST	Positive	25	5.441	0.020
	Negative	31		
	Total	27		
Low-grade NMIBC intervention group				
TDT	Positive	16	6.086	0.014
	Negative	27		
	Total	19		
ST	Positive	27	6.000	0.014
	Negative	38		
	Total	30		

TDT—tumor-doubling time; ST—survival time.

**Table 4:** Univariate Cox regression models for the association between FGFR3 expression, *FGFR3* mutations rate, p53 protein expression, and survival time in control and interventional groups with low-grade GATA3(−)/CR5/6(−) high-PD-L1(+) recurrent NMIBC.

Variables	Control Group		Interventional Group	
	HR (95% CI)	*p* Value	HR (95% CI)	*p* Value
FGFR3 expression	1.299 (0.808–2.087)	0.280	1.380 (0.825–2.307)	0.219
*FGFR3* mutations	4.109 (0.369–45.773)	0.251	4.165 (0.376–46.160)	0.245
p53 expression	0.037 (0.002–0.770)	0.033	0.036 (0.002–0.739)	0.031

## Data Availability

The data presented in this study are available on request from the corresponding author.

## Appendix B

**Table A1:** Patients’ main gender characteristic and clinicopathological features of the tumors.

Sample No	Tumor Stage and Grade	Gender	Age	Histology	PDX
01	Ta, Low grade	Female	71	UPC	Established
02	T1, High grade	Male	63	UPC	Established
03	T1, High grade	Female	71	UPC	Established
04	T1, Low grade	Female	58	UPC	Established
05	T1, High grade	Male	70	UPC	Established
06	T1, Low grade	Male	53	UPC	Not established
07	T1, High grade	Male	59	UPC	Established
08	Ta, Low grade	Female	65	UPC	Established
09	T1, Low grade	Male	73	UPC	Established
10	T1, High grade	Female	58	UPC	Established
11	T1, High grade	Male	48	MPC	Not established
12	T1, Low grade	Male	70	UPC	Established
13	T1, High grade	Male	64	UPC	Established
14	T1, Low grade	Female	59	UPC	Established
15	Ta, Low grade	Female	72	UPC	Established
16	T1, High grade	Male	72	UPC	Established
17	T1, High grade	Female	67	SC	Established
18	T1, Low grade	Male	55	UPC	Established
19	T1, Low grade	Female	68	UPC	Established
20	T1, High grade	Male	46	MPC	Established
21	T1, High grade	Female	59	UPC	Not established
22	Ta, Low grade	Male	61	UPC	Established
23	T1, Low grade	Male	76	UPC	Established
Total	High grade (11) Low grade (12)	Female (10) Male (13)	Average (Mean ± SD) 63.2 ± 2.4	UPC (20) MPC (2) SC (1)	Established (20) Not established (3)

UPC—urothelial papillary carcinoma; SC—squamous cancer; MPC—micropapillary carcinoma.
